# Mechanical Performance of Jute Fiber-Reinforced Micaceous Clay Composites Treated with Ground-Granulated Blast-Furnace Slag

**DOI:** 10.3390/ma12040576

**Published:** 2019-02-14

**Authors:** Jiahe Zhang, Amin Soltani, An Deng, Mark B. Jaksa

**Affiliations:** School of Civil, Environmental and Mining Engineering, The University of Adelaide, Adelaide, SA 5005, Australia; Jiahe.Zhang@adelaide.edu.au (J.Z.); Mark.Jaksa@adelaide.edu.au (M.B.J.)

**Keywords:** micaceous clay, jute fibers, ground-granulated blast-furnace slag, unconfined compression, strength, stiffness, scanning electron microscopy, multivariable regression

## Abstract

The combined capacity of Jute Fibers (JF), the reinforcement, and Ground-Granulated Blast-Furnace Slag (GBFS), the binder, was examined as a sustainable solution towards ameliorating the inferior engineering properties of micaceous clays. A total of sixteen JF + GBFS mix designs, i.e., JF (% by total mass) = {0, 0.5, 1.0, 1.5} and GBFS (% by total mass) = {0, 3, 6, 9}, were tested for unconfined compression (UC) strength; for those mix designs containing GBFS, curing was allowed for 7 and 28 days prior to testing. Scanning electron microscopy (SEM) studies were also carried out to observe the evolution of fabric in response to JF, GBFS and JF + GBFS amendments. The greater the JF content the higher the developed strength and stiffness up to 1% JF, beyond of which the effect of JF-reinforcement led to some adverse results. The JF inclusions, however, consistently improved the ductility and toughness of the composite. The addition of GBFS to the JF-reinforced samples improved the soil–fiber connection interface, and thus led to further improvements in the composite’s strength, stiffness and toughness. The mix design “1% JF + 9% GBFS” managed to satisfy ASTM’s strength criterion and hence was deemed as the optimum choice in this investigation. Finally, a non-linear, multivariable regression model was developed and validated to quantify the peak UC strength as a function of the composite’s index properties. The proposed model contained a limited number of fitting parameters, all of which can be calibrated by little experimental effort, and thus implemented for preliminary design assessments.

## 1. Introduction

Soils are the most common and readily accessible of all materials encountered in construction operations. Most soils, however, are characterized as problematic, as their intrinsic mechanical features, e.g., strength and bearing capacity, are often less than ideal for common civil engineering applications [1,2]. Meanwhile, shortage of land for development, as well as increasing costs associated with construction and raw materials, necessitates maximum utilization of local materials, one being problematic soils; among others, micaceous soils have been less publicized and hence demand further attention. The mica group of sheet silicates are among the most widely distributed minerals around the world; they naturally occur in igneous, sedimentary and certain metamorphic rocks [3,4]. Common physical features of mica include its unique platy structure, high elasticity (owing to its soft, spongy fabric) and nearly perfect basal cleavage; the latter, the nearly perfect cleavage, is attributed to the hexagonal sheet-like arrangement of mica atoms [5,6]. The presence of excessive mica minerals such as muscovite in weathered soils, particularly sands, adversely influence the soil’s mechanical properties. Mica minerals, although rather resilient, may gradually recover their initial shape due to the elastic rebound (or springy action), thereby reducing the efficiency of compactive effort and hence compromising the performance of facilities founded on micaceous soils [7]. During loading, i.e., compression, tension or shearing, mica minerals tend to rotate and orient themselves in a somewhat parallel fashion, which in turn leads to low strength resistance in micaceous soils [8]. Therefore, micaceous soils are characterized by poor compactibility, high compressibility and low shear strength, all of which present significant challenges for road construction, building foundations, earth dams and other geotechnical engineering systems [9,10,11,12,13,14,15,16,17,18,19,20,21]. Consequently, micaceous soils demand engineering solutions to alleviate the associated socio-economic impacts on human life.

Common solutions to counteract the adversities associated with problematic soils, and most likely micaceous clays, include soil replacement or attempting to amend the low-graded soil by means of stabilization [22]. The former involves replacing a portion of the problematic host soil with suitable quarried/burrowed materials capable of satisfying the desired mechanical performance; this approach is often impractical due to long-haul distances, as well as other economic considerations [23]. The latter, soil stabilization, refers to any chemical, physical, biological or combined practice of altering the soil fabric to meet the intended engineering criteria [24]. The chemical stabilization scheme makes use of chemical binders and/or additives—Portland cements, limes, fly ashes and slags, and more recently non-conventional agents such as polymers, resins and sulfonated oils—which initiate a series of short- and long-term chemical reactions in the soil–water medium, thereby amending the soil fabric into a coherent matrix of improved mechanical performance [23,25,26,27,28,29,30,31,32,33,34,35,36,37]. Physical stabilization often involves the placement of random or systematically-engineered reinforcements in the soil regime, thus engendering a spatial three-dimensional reinforcement network in favor of weaving/interlocking the soil particles into a unitary mass of induced strength resistance and improved ductility. Common reinforcements include fibers and geogrids of natural (e.g., bamboo, coir, hemp, jute and sisal) or synthetic (e.g., nylon, polyester, polyethylene, polypropylene and steel) origin, and more recently other sustainable geosynthetics such as waste textiles and recycled tire rubbers, all of which have been well documented in the literature [22,38,39,40,41,42,43,44,45,46,47,48,49,50]. Recent studies indicate that the use of chemical agents, particularly cementitious binders such as Portland cement and lime, alongside physical reinforcements may significantly improve the soil–reinforcement connection interface or bonding, thereby promoting further fabric enhancements [1,51,52,53,54,55,56,57,58,59].

A sustainable soil stabilization scheme can be characterized as one that maintains a perfect balance between infrastructure performance and the social, economic and ecological processes required to maintain human equity, diversity, and the functionality of natural systems. Traditional stabilization agents including cementitious binders and synthetic reinforcements, although proven effective, are not financially competitive in terms of materials procurement, labor and equipment usage. Furthermore, these solutions often suffer from serious environmental drawbacks attributed to their significant energy and carbon emissions footprints [22,60]. As such, the transition towards sustainable soil stabilization necessitates utilizing natural reinforcements and/or industrial by-products as part of the infrastructure system, and more specifically as replacements for traditional stabilization materials. Although the adverse effects of mica content on soils, particularly gravels and sands, have been well documented in the literature, systematic stabilization studies on micaceous soils, and micaceous clays in particular, are still limited [12,61,62,63]. More importantly, the adopted stabilization materials have been limited to Portland cement and lime, while sustainable agents commonly practiced for other problematic soils, e.g., natural fibers and industrial by-products such as fly ashes and slags, have not yet been examined and hence demand further attention.

The present study examines the combined capacity of Jute Fibers (JF), the reinforcement, and Ground-Granulated Blast-Furnace Slag (GBFS), the binder, as a sustainable solution towards ameliorating the inferior engineering characteristics of micaceous clays. A series of unconfined compression (UC) tests were carried out on various mix designs to evaluate the effects of JF-reinforcement and/or GBFS-treatment on the strength, ductility, stiffness and toughness of the micaceous clay. Scanning electron microscopy (SEM) studies were also carried out to observe the evolution of soil fabric in response to JF, GBFS and JF + GBFS amendments. Finally, a non-linear, multivariable regression model was developed and validated to quantify the peak UC strength as a function of the composite’s index properties. A sensitivity analysis was also carried out to quantify the relative impacts of the independent regression variables, namely JF content, GBFS content and curing time, on the composite’s strength.

## 2. Materials

### 2.1. Micaceous Clay

Commercially-available Kaolin (K) and Ground Mica (GM), sourced from local distributors, were used to artificially prepare a desired Micaceous Clay (MC) blend for further experimental work. The choice of GM content for the MC blend was selected as 20% (by dry mass of K), as it represents an upper boundary prerequisite to simulate adverse mechanical attributes commonly exhibited by natural micaceous clays, i.e., compactability issues and low shear strength/bearing capacity [10,11,16]. The artificial MC blend manifested the same typical texture, sheen and friability features as natural micaceous clays commonly reported in the literature, and thus may well provide a basis for systematic stabilization studies. The physical and mechanical properties of K, GM and the MC blend (hereafter simply referred to as natural soil) were determined as per relevant ASTM and Australian (AS) standards, and the results are summarized in Table 1. The conventional gradation analysis, carried out in accordance with ASTM D422–07, indicated a clay fraction (<2 μm) of 51%, along with 48% silt (2–75 μm) and 1% sand (0.075–4.75 mm) for K. As a result of 20% GM inclusion, the aforementioned values changed to 39%, 55% and 6%, respectively. The liquid limit and plasticity index were measured as LL = 44.67% and PI = 20.95% for K, and LL = 48.67% and PI = 11.28% for MC, from which these soils were, respectively, characterized as *clay with intermediate plasticity* (CI) and *silt with intermediate plasticity* (MI) in accordance with the Unified Soil Classification System (USCS). The standard Proctor compaction test (ASTM D698–12) indicated optimum water contents of *w*_opt_ = 19.84% and 23.52%, along with maximum dry densities of *ρ*_dmax_ = 1.63 g/cm^3^ and 1.56 g/cm^3^, for K and MC, respectively. Such trends can be attributed to the spongy nature (i.e., elastic/rebound response to compaction energy) and high water demand of the mica mineral [12,20,64].

The chemical compositions of K and GM, as supplied by the manufacturers, are outlined in Table 2. The chemical composition of both K and GM is mainly dominated by silicon dioxide (SiO_2_) and aluminum trioxide (Al_2_O_3_) with mass fractions of 64.9% and 22.2% for K, and 49.5% and 29.2% for GM, respectively. The pH for slurries of K and GM was, respectively, found to be 7.4 and 7.8, from which both materials were classified as neutral substances. Other material properties included a specific surface area of SSA = 11.2 m^2^/g and 5.3 m^2^/g for K and GM, respectively.

### 2.2. Jute Fibers

**Figure 1:**
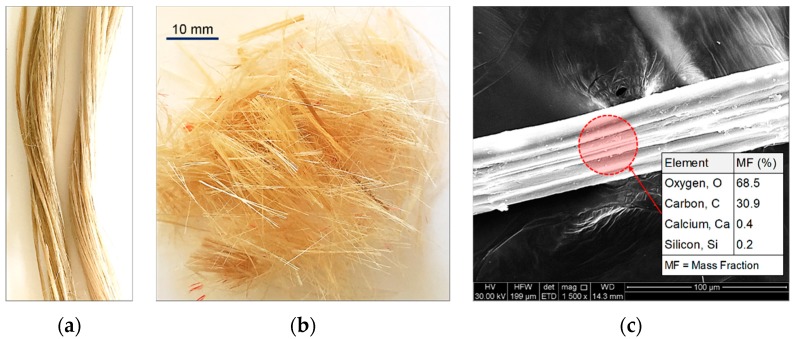
JF at different magnification ratios: (**a**) Raw fibers (no magnification); (**b**) Processed fibers (no magnification); and (**c**) Processed fibers (1500× magnification).

Commercially-available Jute Fibers (JF), manufactured from *Corchorus capsularis* (a shrub species in the Malvaceae family), was used as the reinforcing agent. Its biochemical composition, as commonly reported in the literature, consists of 56–71% cellulose, 29–35% hemicellulose and 11–14% lignin [66]. The raw fibers had a diameter of *F*_D_ = 30–40 μm; they were cut into segments of approximately *F*_L_ = 15 mm, thus resulting in an aspect ratio of *F*_AR_ = *F*_L_/*F*_D_ = 375–500 (see Figure 1a,b). The scanning electron microscopy (SEM) technique was used to observe the fiber’s surface morphology, and the results are illustrated in Figure 1c. The fiber’s surface embodies a highly-irregular shape comprising of a series of peaks and troughs of varying heights, depths and spacing, thus signifying a rough surface texture. Such surface features may potentially promote adhesion and/or induce frictional resistance at the soil–fiber interface, and thus amend the soil fabric into a coherent matrix of induced strength and improved ductility (see Section 4.3). The physical and mechanical properties of JF, as supplied by the distributor, are provided in Table 3. The specific gravity of JF was found to be 1.30–1.46, which is approximately two-fold less than that of the MC blend.

### 2.3. Ground-Granulated Blast-Furnace Slag

A large quantity of Ground-Granulated Blast-Furnace Slag (GBFS) was sourced from a local manufacturer in South Australia, and was used as the cementitious binder. The physical properties and chemical composition of GBFS, as supplied by the manufacturer, are outlined in Table 4. The particles of GBFS were mainly finer than 75 μm in size; its fines and sand fractions were found to be 96% and 4%, respectively. Other properties included a basic pH of 9.6 and a specific surface area of SSA = 0.7 m^2^/g; the latter is approximately two-fold greater than that of ordinary Portland cement [67]. The chemical composition of GBFS is mainly dominated by calcium oxide or lime (CaO) and silicon dioxide (SiO_2_) with mass fractions of 44.7% and 27.1%, respectively. The former, the calcium oxide, acts as a precursor agent, initiating a series of short- and long-term chemical reactions in the soil–water medium, i.e., cation exchange, flocculation–agglomeration and pozzolanic reactions, thereby amending the soil fabric into a unitary mass of enhanced mechanical performance (see Section 4.3).

## 3. Experimental Program

### 3.1. Mix Designs and Sample Preparations

In this study, a total of sixteen mix designs consisting of one control (natural soil), three JF-reinforced, three GBFS-treated and nine JF + GBFS blends were examined (see Table 5). Hereafter, the following coding system is adopted to designate the various mix designs:

(1) $$F_{x}S_{y}T_{z}$$

where *F_x_* = *x*% JF; *S_y_* = *y*% GBFS; and *T_z_* = *z* days of curing.

The JF, GBFS and water contents were, respectively, defined as:

(2) $$(\%)\;F_{c}=\frac{m_{\mathrm{JF}}}{m_{\mathrm{JF}}+m_{\mathrm{GBFS}}+m_{\mathrm{MC}}}\times 100$$

(3) $$(\%)\;S_{c}=\frac{m_{\mathrm{GBFS}}}{m_{\mathrm{GBFS}}+m_{\mathrm{JF}}+m_{\mathrm{MC}}}\times 100$$

(4) $$(\%)\;w_{c}=\frac{m_{W}}{m_{\mathrm{JF}}+m_{\mathrm{GBFS}}+m_{\mathrm{MC}}}\times 100$$

where *F*_c_ = JF content; *S*_c_ = GBFS content; *w*_c_ = water content; *m*_JF_ = mass of JF; *m*_GBFS_ = mass of GBFS; *m*_MC_ = mass of micaceous clay (or natural soil); and *m*_W_ = mass of water.

The natural soil, JF and GBFS were blended in dry form as per the selected mix designs outlined in Table 5. Mixing was carried out for approximately 5 min to gain visible homogeneity of the ingredients. The required volume of water corresponding to a water content of *w*_c_ = 23.52%, the standard Proctor optimum water content of the natural soil (ASTM D698–12), was added to each blend and thoroughly mixed by hand for approximately 15 min. Extensive care was taken to pulverize the clumped particles, targeting homogeneity of the mixtures. A special split mold, similar to that described in the literature, was designed and fabricated from stainless steel to accomplish static compaction [33,43,49]. The mold consisted of three segments, namely the top collar, the middle section, and the bottom collar. The middle section measures 50 mm in diameter and 100 mm in height, and accommodates the sample for the unconfined compression test (see Section 3.2). The moist blends were statically compacted in the mold in five layers; each layer achieved a dry density of *ρ*_d_ = 1.56 g/cm^3^ (i.e., the standard Proctor maximum dry density of the natural soil, obtained as per ASTM D698–12). The surface of the first to fourth compacted layers was scarified to ensure adequate bonding between adjacent layers of the mixture. Samples containing GBFS were enclosed in multiple layers of cling wrap and transferred to a humidity chamber, maintained at 70% relative humidity and a temperature of 25 ± 2 °C, where curing was allowed for 7 and 28 days prior to testing.

**Figure 2:**
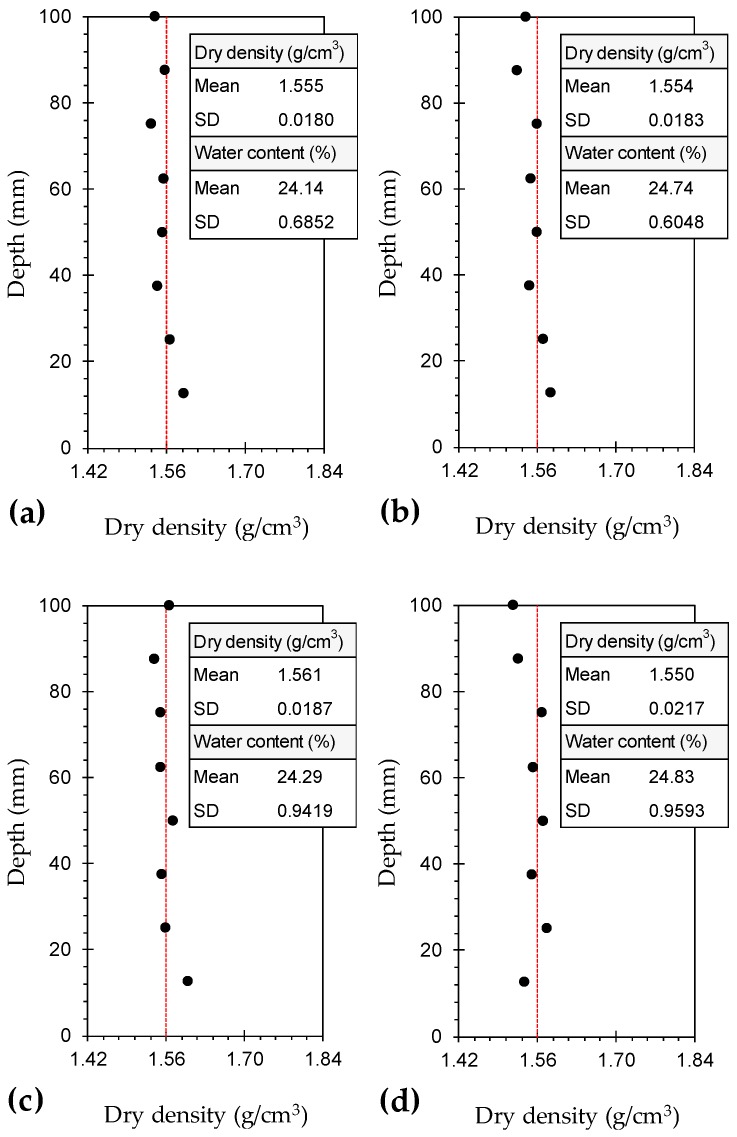
Variations of dry density along the height of the compacted samples: (**a**) *F*_0_*S*_0_*T*_0_; (**b**) *F*_1.0_*S*_0_*T*_0_; (**c**) *F*_0_*S*_6_*T*_0_; and (**d**) *F*_1.0_*S*_6_*T*_0_.

To ensure uniformity of fabric and hence consistency in behavior, the variations of dry density and water content should be measured along the height of the compacted samples [68]. In this regard, typical cases including *F*_0_*S*_0_*T*_0_ (natural soil), *F*_1.0_*S*_0_*T*_0_, *F*_0_*S*_6_*T*_0_ and *F*_1.0_*S*_6_*T*_0_ were examined, and the results are provided in Figure 2. The variations of both dry density and water content were found to be marginal, as evident with the low standard deviations (SD), thus corroborating the suitability of the adopted sample preparation technique.

### 3.2. Unconfined Compression Test

Unconfined compression (UC) tests were carried out in accordance with ASTM D2166–16. The prepared samples (see Section 3.1) were axially compressed at a constant displacement rate of 1 mm/min (equivalent to 1%/min), as commonly adopted in the literature [22,33,69]. Axial strains and the corresponding axial stresses were recorded at various time intervals to a point at which the maximum axial stress required for sample failure, denoted as the peak UC strength, was achieved. On account of the two curing times adopted for the samples containing GBFS, a total of 28 UC tests, i.e., one for control (natural soil), three for JF-reinforced, six for GBFS-treated and eighteen for JF + GBFS blends, were conducted to address the sixteen mix designs outlined in Table 5. To ensure sufficient accuracy, triplicate samples were tested for typical mix designs, i.e., *F*_0_*S*_0_*T*_0_ (natural soil), *F*_1.0_*S*_0_*T*_0_, *F*_0_*S*_6_*T*_28_ and *F*_1.0_*S*_6_*T*_28_. In this regard, the standard deviation (SD) and the coefficient of variation (CV) for the triplicate peak UC strength data were found to range between SD = 3.74 kPa and 11.19 kPa, and CV = 3.23% and 5.15%; these low values corroborate the repeatability of the adopted sample preparation technique, as well as the implemented UC testing procedure.

### 3.3. Scanning Electron Microscopy Studies

The scanning electron microscopy (SEM) technique was implemented to investigate the evolution of fabric in response to JF, GBFS and JF + GBFS amendments. SEM imaging was carried out by means of the Philips XL20 (Amsterdam, The Netherlands) scanning electron microscope. Apparatus specifications included a resolution of 4 μm and a maximum magnification ratio of 50,000×. In this regard, typical mix designs consisting of *F*_0_*S*_0_*T*_0_ (natural soil), *F*_1.0_*S*_0_*T*_0_, *F*_0_*S*_6_*T*_28_ and *F*_1.0_*S*_6_*T*_28_ were examined. The desired samples, prepared as per Section 3.1, were first air-dried for approximately 14 days. The desiccated samples were then carefully fractured into small cubic-shaped pieces measuring approximately 1000 mm^3^ in volume, and were further subjected to SEM imaging at various magnification ratios ranging from 250× to 20,000×.

## 4. Results and Discussion

### 4.1. Effect of JF on UC Strength

**Figure 3:**
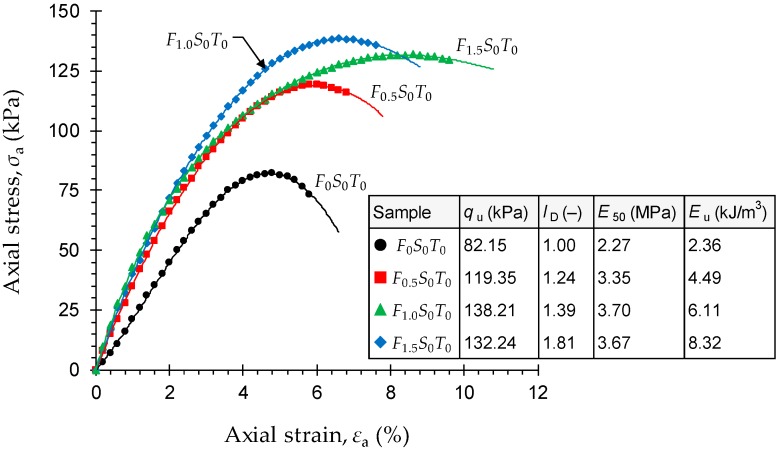
Stress–strain curves for the natural soil and various JF-reinforced samples, i.e., *F_x_S_y_T_z_* where *x* = {0, 0.5, 1.0, 1.5}, *y* = {0}, and *z* = {0}.

Stress–strain curves for the natural soil and various JF-reinforced samples—*F_x_S_y_T_z_* where *x* = {0, 0.5, 1.0, 1.5}, *y* = {0}, and *z* = {0}—are provided in Figure 3. The stress–strain relationship for the natural soil sample demonstrated a rise–fall response with a visually-detectable peak point, thereby indicating a strain-softening behavior accompanied by a brittle sample failure. As a result of JF-reinforcement, the stress–strain locus progressively transitioned towards a strain-hardening character. In this case, the greater the JF content the more prominent the strain-hardening effect and hence the less dramatic (or the more ductile) the failures.

As demonstrated in Figure 3, the greater the JF content the higher the peak UC strength up to *F*_c_ = 1%, beyond of which JF-reinforcement was found to adversely influence strength development in the composite. The natural soil exhibited a peak UC strength of *q*_u_ = 82.15 kPa, while the samples reinforced with *F*_c_ = 0.5% and 1% resulted in higher values of *q*_u_ = 119.35 kPa and 138.21 kPa, respectively. The higher JF inclusion of 1.5% changed the peak UC strength to 132.24 kPa, which still holds a notable advantage over the natural soil, as well as the sample reinforced with 0.5% JF. The axial strain at failure, denoted as *ε*_u_, is an indication of the material’s ductility; higher *ε*_u_ values manifest a more ductile (or a less brittle) character. Improvement in ductility is often quantified by means of the deformability index *I*_D_ [70]:

(5) $$I_{D}=\frac{{\varepsilon_{u}}^{S}}{{\varepsilon_{u}}^{N}}$$

where *ε*_u_^S^ = axial strain at failure for the stabilized soil sample; and *ε*_u_^N^ = axial strain at failure for the control (or natural soil) sample.

The deformability index exhibited a monotonically-increasing trend with JF content, thus indicating that the greater the JF content the more ductile the sample’s response to compression. By definition, the natural soil corresponds to a deformability index of unity (*ε*_u_^N^ = 4.73%). As a result of JF-reinforcement, the deformability index exhibited a monotonically-increasing trend, and resulted in *I*_D_ = 1.24, 1.39 and 1.81 (*ε*_u_^S^ = 5.88%, 6.57% and 8.55%) for *F*_c_ = 0.5%, 1% and 1.5%, respectively.

The secant modulus at 50% of the peak UC strength, denoted as *E*_50_, is a measure of the material’s stiffness in the elastic compression domain [22,71]. The variations of *E*_50_, as given in Figure 3, exhibited a trend similar to that observed for the peak UC strength, peaking at *F*_c_ = 1% and then slightly decreasing for the higher JF content of 1.5%. The natural soil and samples reinforced with 0.5%, 1% and 1.5% JF resulted in *E*_50_ = 2.27 MPa, 3.35 MPa, 3.70 MPa and 3.67 MPa, respectively. The area under a typical stress–strain curve up to the peak point, defined as the energy stored by a sample undergoing deformation and referred to as peak strain energy, serves as a measure of the material’s toughness [22,72]. Unlike strength and stiffness, the development of toughness, similar to ductility, was consistently in favor of the JF inclusions, and displayed a monotonically-increasing trend with respect to JF content (see the *E*_u_ values in Figure 3). An increase in toughness warrants an increase in the peak UC strength and/or the axial strain at failure [41,57]. With regard to JF-reinforcement, both *q*_u_ and *ε*_u_ contribute to the development of toughness; however, the greater the JF content the less prominent the strength’s contribution and hence the more significant the role of ductility. The natural soil resulted in *E*_u_ = 2.36 kJ/m^3^, while the samples reinforced with *F*_c_ = 0.5%, 1% and 1.5% resulted in higher values of *E*_u_ = 4.49 kJ/m^3^, 6.11 kJ/m^3^ and 8.32 kJ/m^3^, respectively.

### 4.2. Effect of JF + GBFS on UC Strength

**Figure 4:**
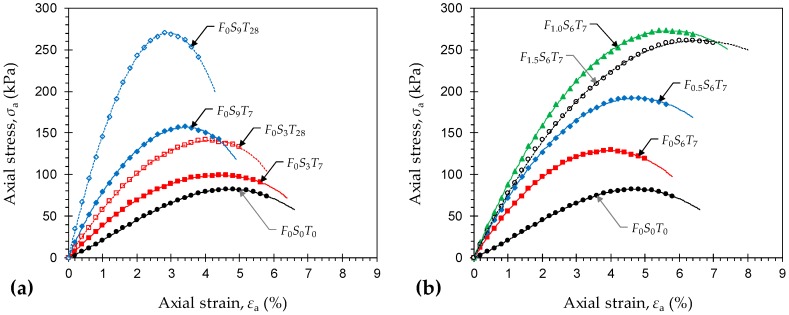
Typical stress–strain curves for the natural soil (*F*_0_*S*_0_*T*_0_) and various stabilized samples: (**a**) *F_x_S_y_T_z_* where *x* = {0}, *y* = {3, 9}, and *z* = {7, 28}; and (**b**) *F_x_S_y_T_z_* where *x* = {0, 0.5, 1.0, 1.5}, *y* = {6}, and *z* = {7}.

Typical stress–strain curves for the natural soil (*F*_0_*S*_0_*T*_0_) and various GBFS-treated samples—*F_x_S_y_T_z_* where *x* = {0}, *y* = {3, 9}, and *z* = {7, 28}—are provided in Figure 4a. Unlike the JF-reinforced samples (see Figure 3), the stress–strain responses for all GBFS-treated composites were seemingly strain-softening and hence accompanied by brittle failures. In general, the greater the GBFS content and/or the longer the curing period, the higher the developed strength and stiffness, and the more prominent the strain-softening character. Stress–strain curves for the natural soil (*F*_0_*S*_0_*T*_0_) and various JF-reinforced samples treated with 6% GBFS—*F_x_S_y_T_z_* where *x* = {0, 0.5, 1.0, 1.5}, *y* = {6}, and *z* = {7}—are provided in Figure 4b. Much like the natural soil reinforced with JF (see Figure 3), for any given GBFS content, an increase in JF content progressively transitioned the stress–strain locus towards a strain-hardening character. In this case, the greater the JF content the more pronounced the strain-hardening effect and hence the more ductile the failures.

**Figure 5:**
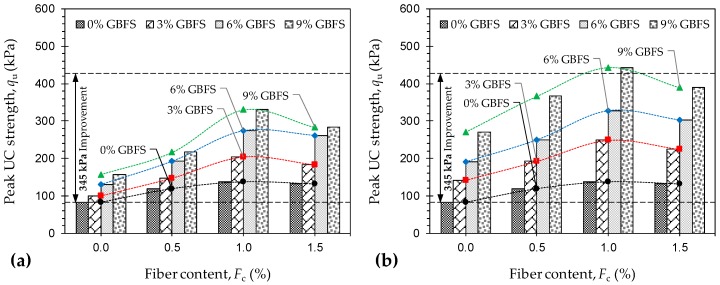
Variations of peak UC strength *q*_u_ against JF content for the natural soil and various GBFS-treated samples: (**a**) *T*_c_ = 7 days; and (**b**) *T*_c_ = 28 days.

Figure 5a,b illustrate the variations of peak UC strength against JF content for the natural soil and various GBFS-treated samples tested at 7 and 28 days of curing, respectively. Much like the natural soil reinforced with JF, for any given GBFS content and curing time, the peak UC strength increased with JF content up to *F*_c_ = 1%; beyond 1% JF, the effect of JF-reinforcement adversely influenced strength development in the composite. For instance, the sample *F*_0_*S*_6_*T*_28_ resulted in *q*_u_ = 191.32 kPa, while the inclusions of 0.5%, 1% and 1.5% JF, with the same 6% GBFS content and the same 28-day curing condition, resulted in *q*_u_ = 250.08 kPa, 327.42 kPa and 302.76 kPa, respectively. Moreover, for any given JF content, the greater the GBFS content and/or the longer the curing period, the higher the developed peak UC strength, following a monotonically-increasing trend. The sample *F*_1.0_*S*_0_*T*_0_, for instance, exhibited a peak UC strength of *q*_u_ = 138.21 kPa. As a result of 3%, 6% and 9% GBFS inclusions, along with the same 1% JF content and a 7-day curing condition, the peak UC strength increased to 203.56 kPa, 273.68 kPa and 330.06 kPa, respectively. Similar mix designs cured for *T*_c_ = 28 days exhibited significant improvements over their 7-day counterparts, as the aforementioned values increased to 248.65 kPa, 327.42 kPa and 443.21 kPa, respectively. The ASTM D4609–08 standard suggests a minimum improvement of 345 kPa in the natural soil’s peak UC strength (at *T*_c_ = 28 days) as a criterion for characterizing an effective stabilization scheme [34]. As demonstrated in Figure 5b, the sample *F*_1.0_*S*_9_*T*_28_ promotes a 361.06 kPa improvement in the peak UC strength and hence satisfies the aforementioned criterion.

**Figure 6:**
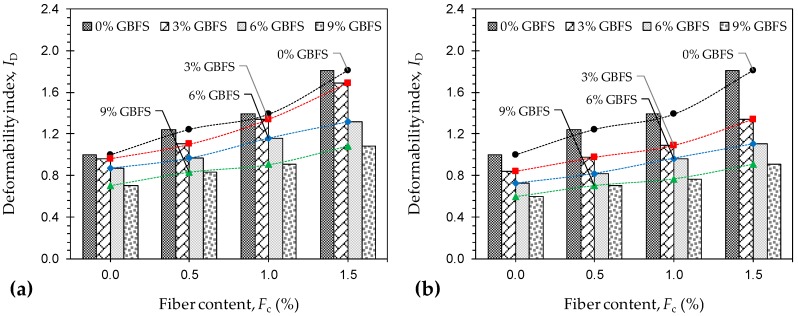
Variations of deformability index *I*_D_ against JF content for the natural soil and various GBFS-treated samples: (**a**) *T*_c_ = 7 days; and (**b**) *T*_c_ = 28 days.

The deformability index, a measure of the material’s ductility, was also calculated for various JF + GBFS mix designs, and the results are provided in Figure 6a,b for the samples tested at *T*_c_ = 7 and 28 days, respectively. Similar to the natural soil reinforced with JF, for any given GBFS content and curing time, the greater the JF content the higher the deformability index, following a monotonically-increasing trend. For any given JF content, however, the greater the GBFS content and/or the longer the curing period, the lower the developed ductility. The deformability index for various JF + GBFS blends was cross-checked with that of the natural soil (or *I*_D_ = 1) to arrive at the optimum cases. In this regard, nine cases (out of 28) manage to satisfy the *I*_D_ ≥ 1 criterion, and thus are deemed as optimum with respect to ductility improvement. The nine optimum cases and their corresponding *I*_D_ values include *F*_0.5_*S*_3_*T*_7_ (*I*_D_ = 1.10), *F*_1.0_*S*_3_*T*_7_ (*I*_D_ = 1.34), *F*_1.5_*S*_3_*T*_7_ (*I*_D_ = 1.68), *F*_1.0_*S*_3_*T*_28_ (*I*_D_ = 1.09), *F*_1.5_*S*_3_*T*_28_ (*I*_D_ = 1.34), *F*_1.0_*S*_6_*T*_7_ (*I*_D_ = 1.16), *F*_1.5_*S*_6_*T*_7_ (*I*_D_ = 1.32), *F*_1.5_*S*_6_*T*_28_ (*I*_D_ = 1.10), and *F*_1.5_*S*_9_*T*_7_ (*I*_D_ = 1.08).

**Figure 7:**
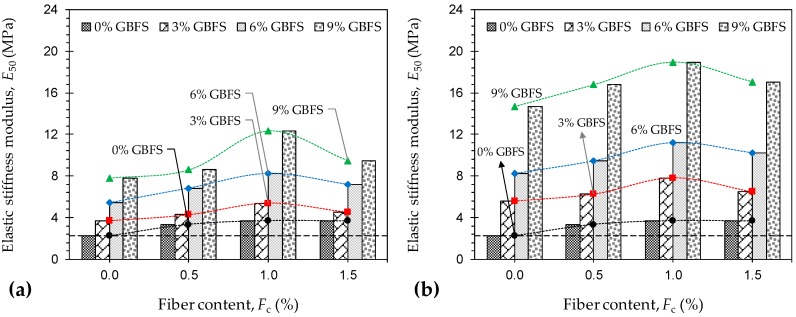
Variations of *E*_50_ against JF content for the natural soil and various GBFS-treated samples: (**a**) *T*_c_ = 7 days; and (**b**) *T*_c_ = 28 days.

Figure 7a,b illustrate the variations of *E*_50_ against JF content for the natural soil and various GBFS-treated samples tested at 7 and 28 days of curing, respectively. The variations of *E*_50_ exhibited a trend similar to that observed for the peak UC strength given in Figure 5. As such, for any given JF content, the development of stiffness was in favor of both the GBFS content and the curing time. As typical cases, the samples *F*_1.0_*S*_0_*T*_0_, *F*_1.0_*S*_3_*T*_7_, *F*_1.0_*S*_3_*T*_28_, *F*_1.0_*S*_9_*T*_7_ and *F*_1.0_*S*_9_*T*_28_ resulted in *E*_50_ = 3.70 MPa, 5.39 MPa, 7.81 MPa, 12.30 MPa and 18.92 MPa, respectively. Moreover, for any given GBFS content and curing time, stiffness enhancements were only notable for samples with up to 1% JF inclusions. In this regard, the samples *F*_0_*S*_6_*T*_28_, *F*_0.5_*S*_6_*T*_28_, *F*_1.0_*S*_6_*T*_28_ and *F*_1.5_*S*_6_*T*_28_, for instance, resulted in *E*_50_ = 8.25 MPa, 9.47 MPa, 11.21 MPa and 10.23 MPa, respectively.

**Figure 8:**
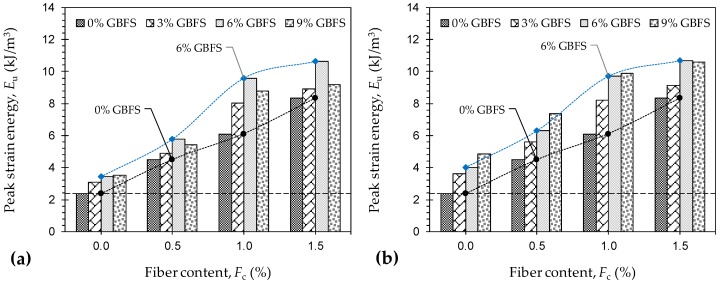
Variations of peak strain energy *E*_u_ against JF content for the natural soil and various GBFS-treated samples: (**a**) *T*_c_ = 7 days; and (**b**) *T*_c_ = 28 days.

Figure 8a,b illustrate the variations of peak strain energy, a measure of the material’s toughness, against JF content for the natural soil and various GBFS-treated samples tested at 7 and 28 days of curing, respectively. The development of toughness was in favor of both the JF content and the GBFS treatments (i.e., GBFS content and/or curing time). For any given GBFS content and curing time, the greater the JF content the higher the peak strain energy, following a monotonically-increasing trend. For instance, the samples *F*_0_*S*_6_*T*_28_, *F*_0.5_*S*_6_*T*_28_, *F*_1.0_*S*_6_*T*_28_ and *F*_1.5_*S*_6_*T*_28_ resulted in peak strain energies of *E*_u_ = 3.99 kJ/m^3^, 6.30 kJ/m^3^, 9.71 kJ/m^3^ and 10.70 kJ/m^3^, respectively. Similarly, for any given JF content, the greater the GBFS content and/or the longer the curing period, the higher the developed toughness. As typical cases, the sample *F*_1.0_*S*_0_*T*_0_ resulted in *E*_u_ = 6.11 kJ/m^3^, while the aforementioned value increased to 8.02 kJ/m^3^, 8.22 kJ/m^3^, 8.78 kJ/m^3^ and 9.88 kJ/m^3^ for *F*_1.0_*S*_3_*T*_7_, *F*_1.0_*S*_3_*T*_28_, *F*_1.0_*S*_9_*T*_7_ and *F*_1.0_*S*_9_*T*_28_, respectively.

**Figure 9:**
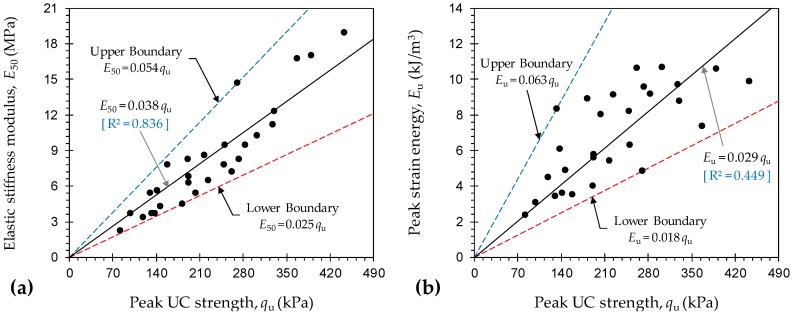
Variations of (**a**) *E*_50_ and (**b**) peak strain energy *E*_u_ against peak UC strength *q*_u_ for various JF + GBFS blends.

Figure 9a,b illustrate the variations of *E*_50_ and *E*_u_ against *q*_u_ for various JF + GBFS mix designs, respectively. The variations of *E*_50_ were situated within the 0.054*q*_u_ < *E*_50_ < 0.025*q*_u_ domain (*E*_50_ in MPa, and *q*_u_ in kPa). For *E*_u_, however, a broader domain in the form of 0.063*q*_u_ < *E*_u_ < 0.018*q*_u_ (*E*_u_ in kJ/m^3^, and *q*_u_ in kPa) was noted. The former, the *E*_50_, exhibited a rather strong correlation with *q*_u_. On the contrary, the peak strain energy was poorly correlated with the peak UC strength. In this regard, simple correlative models in the forms of *E*_50_ = 0.038*q*_u_ (with R^2^ = 0.836) and *E*_u_ = 0.029*q*_u_ (with R^2^ = 0.449) can be derived; the former can be implemented for indirect estimations of *E*_50_.

### 4.3. Stabilization Mechanisms and Microstructure Analysis

The JF inclusions are able to amend the soil fabric through improvements achieved in two aspects: (**i**) frictional resistance generated at the soil–fiber interface, owing to the fiber’s rough surface texture; and (**ii**) mechanical interlocking of soil particles and fibers [1,22,40,45,48,51,57,66]. The interfacial frictional resistance is a function of the soil–fiber contact area, with greater contact levels providing a higher resistance to bear the external loads. Consequently, this amending mechanism can be ascribed to the fiber content, meaning that the greater the number of included fiber units, i.e., increase in fiber content, the greater the contact levels achieved between the soil particles and fibers, and thus the higher the generated interfacial frictional resistance against UC loading. The second amending mechanism, the mechanical interlocking of soil particles and fibers, is achieved during sample preparation/compaction, and induces the composite’s adhesion by immobilizing the soil particles undergoing shearing. Quite clearly, the more effective/pronounced the achieved mechanical interlocking the higher the permanence against UC loading. Consequently, this amending mechanism is in line with the fiber content, and more importantly the fiber’s elongated form factor. In general, the greater the number of included fiber units, i.e., increase in fiber content, the greater the number of interlocked or enwrapped soil aggregates, and thus the higher the developed peak UC strength. It should be noted that the soil–fiber amending mechanisms, as described above, only hold provided that the fiber units do not cluster (or adhere to each other) during mixture preparation and compaction [22,54,56,73]. At high fiber contents, the behavior of the composite, at some points, may be governed by a dominant fiber-to-fiber interaction; this effect, commonly referred to as fiber-clustering, leads to a notable improvement in the sample’s ductility/deformability and toughness (see Figure 6 and Figure 8) while offsetting the desired soil-to-fiber interaction capable of improving the sample’s peak UC strength and stiffness. Fiber-clustering effects were evident for all samples containing 1.5% JF, as the previously-improved peak UC strength and stiffness manifested a notable decrease compared with similar mix designs containing 1% JF (see Figure 5 and Figure 7).

Calcium-based binders, in this case GBFS, initiate a series of short- and long-term chemical reactions in the soil–water medium, which alter the soil fabric into a unitary mass of improved mechanical performance. Short-term chemical reactions consist of cation exchange and flocculation–agglomeration; their amending roles are often negligible when paired with neutrally-charged soil particles such as gravels, sands and silts. For fine-grained soils containing a notable fraction of negatively-charged clay particles, however, short-term reactions lead to significant improvements in the soil’s plasticity/workability, early-age strength, swelling potential and consolidation capacity [33,74,75,76]. During short-term reactions, higher-valence cations substitute those of lower valence, and cations of larger ionic radius replace smaller cations of the same valence; the order of substitution follows the Hofmeister (or Lyotropic) series, i.e., Na^+^ < K^+^ << Mg^2+^ < Ca^2+^ [77]. GBFS-treatment supplies the clay–water medium with additional calcium cations (Ca^2+^), which immediately substitute cations of lower valence (e.g., sodium Na^+^) and/or same-valence cations of smaller ionic radius (e.g., magnesium Mg^2+^) in the vicinity of the clay particles. These cation exchanges lead to a decrease in the thickness of the Diffused Double Layers (DDLs), owing to the development of strong van der Waals bonds between adjacent clay particles in the matrix, which in turn promote aggregation and flocculation of the clay particles [76,78,79]. Long-term chemical reactions, commonly referred to as pozzolanic reactions, are strongly time- and often temperature-dependent, meaning that their commencement and evolution require a certain and often long period of curing. During pozzolanic reactions, ionized calcium (Ca^2+^) and hydroxide (OH^–^) units, released from the water–binder complex, gradually react with silicate (SiO_2_) and aluminate (Al_2_O_3_) units in the soil, thereby leading to the formation of strong cementation products/gels, namely Calcium–Silicate–Hydrates (CSH), Calcium–Aluminate–Hydrates (CAH) and Calcium–Aluminate–Silicate–Hydrates (CASH); these products encourage further solidification and flocculation of the soil particles, which in turn accommodate the development of a dense, uniform matrix coupled with enhanced strength performance [31,33,76,79]. It should be noted that the short- and long-term amending reactions, as described above, are generally in favor of a higher binder content; this general perception also complies with the results outlined in Figure 5, Figure 7 and Figure 8.

**Figure 10:**
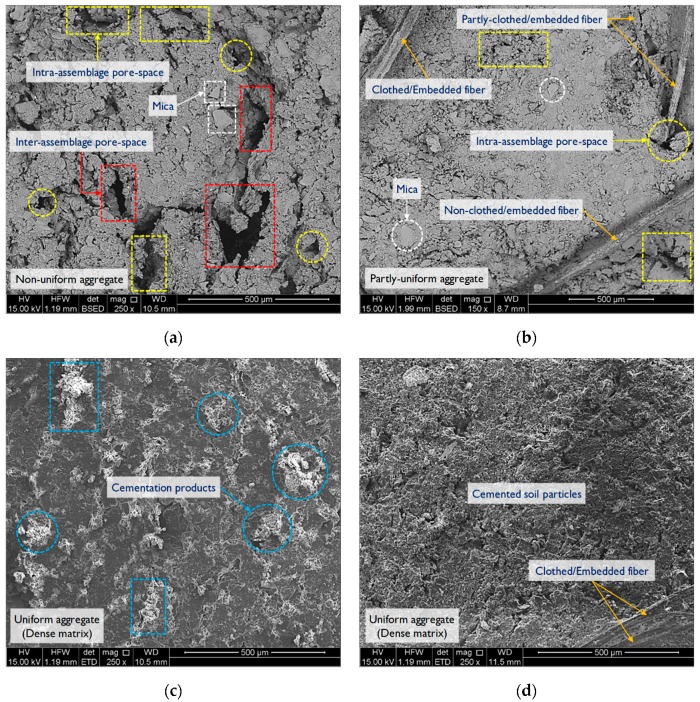
SEM micrographs for the tested samples: (**a**) *F*_0_*S*_0_*T*_0_ (natural soil); (**b**) *F*_1.0_*S*_0_*T*_0_; (**c**) *F*_0_*S*_6_*T*_28_; and (**d**) *F*_1.0_*S*_6_*T*_28_.

The microstructure analysis was carried out using an SEM characterization scheme developed by Soltani et al. [58]. Figure 10a–d illustrate SEM micrographs for the samples *F*_0_*S*_0_*T*_0_ (natural soil), *F*_1.0_*S*_0_*T*_0_, *F*_0_*S*_6_*T*_28_ and *F*_1.0_*S*_6_*T*_28_, respectively. The microstructure of the natural soil sample manifested a partly-dense, non-uniform matrix, accompanied by a notable number of large inter- and intra-assemblage pore-spaces, respectively, formed between and within the soil aggregates; such morphological features warrant the existence of an edge-to-edge dispersed fabric (see Figure 10a). The microstructure of the JF-reinforced sample or *F*_1.0_*S*_0_*T*_0_ exhibited a partly-dense but more uniform matrix, accompanied by a limited number of small intra-assemblage pore-spaces mainly distributed along the soil–fiber connection interface. In essence, the fiber units acted as physical anchors within the matrix, interlocking the neighboring soil aggregates and hence withstanding compressive stresses during shearing (see Figure 10b). As a result of GBFS-treatment (see sample *F*_0_*S*_6_*T*_28_ in Figure 10c), the microstructure became even more uniform in nature, indicating aggregation and flocculation of the soil particles and hence the development of a fully-dense matrix with a dominant edge-to-face flocculated fabric. Prevalent cementation products were clearly visible between and within the soil aggregates, which portrayed a major role in eliminating the inter- and intra-assemblage pore-spaces in the matrix. As a result of JF-reinforcement and GBFS-treatment (see sample *F*_1.0_*S*_6_*T*_28_ in Figure 10d), the soil–fiber connection interface was markedly improved, as evident with the presence of fully-clothed fibers strongly embedded between and within the soil aggregates, which in turn led to a further improvement in the composite’s strength and stiffness.

## 5. Modeling

### 5.1. Model Development

For a given type of soil reinforced with JF and/or treated with GBFS, the independent variables governing the peak UC strength *q*_u_ (in kPa), as evident with the experimental results discussed in Section 4, can be categorized as: (**i**) JF content *F*_c_ (in %); (**ii**) GBFS content *S*_c_ (in %); and (**iii**) curing time *T*_c_ (in days). Therefore, the peak UC strength problem for various JF + GBFS blends can be expressed as:

(6) $$q_{u}=f(F_{c},S_{c},T_{c})$$

where *f* = an unknown functional expression which is to be obtained through trial and error.

A suitable regression model can be characterized as one that maintains a perfect balance between simplicity, i.e., ease of application, and accuracy, i.e., acceptable goodness of fit and low forecast error. As such, any suggested functional expression for *f* should involve a simple algebraic structure, constructed by a minimal number of model/fitting parameters (or regression coefficients), capable of arriving at a reliable estimate of the problem at hand [50,80]. The multivariable quadratic function, as demonstrated in Equation (7) for the JF + GBFS peak UC strength problem, often serves as a suitable starting point to initiate the trial and error stage, and thus identify statistically-meaningful functional components capable of constructing a regression model which is both simple in structure and accurate in terms of predictive capacity [43,55,59,81].

(7) $$q_{u}=\beta_{0}+\beta_{1}F_{c}+\beta_{2}S_{c}+\beta_{3}T_{c}+\beta_{4}{F_{c}}^{2}+\beta_{5}{S_{c}}^{2}+\beta_{6}{T_{c}}^{2}+\beta_{7}F_{c}S_{c}+\beta_{8}F_{c}T_{c}+\beta_{9}S_{c}T_{c}$$

where *β*_0_ to *β*_9_ = model/fitting parameters (or regression coefficients); and *β*_0_ = peak UC strength of the natural soil, since setting *F*_c_ = 0, *S*_c_ = 0 and *T*_c_ = 0 leads to *q*_u_ = *β*_0_.

The model proposed in Equation (7) was fitted to the experimental peak UC strength data (presented in Figure 5) by means of the least-squares optimization technique. Routine statistical tests, namely Fisher’s *F*–test and Student’s *t*–test, were then carried out to examine the model’s statistical significance. In addition, statistical fit-measure indices, such as the coefficient of determination R^2^ (dimensionless), the root-mean-squared error RMSE (in kPa), the normalized root-mean-squared error NRMSE (in %) and the mean-absolute-percentage error MAPE (in %), were adopted to assess the model’s predictive capacity [82,83]:

(8) $$\mathrm{RMSE}=\sqrt{\frac{1}{N}\sum_{b=1}^{N}{\left[{\left({q_{u}}^{A}\right)}_{b}-{\left({q_{u}}^{P}\right)}_{b}\right]}^{\;2}}$$

(9) $$(\%)\;\mathrm{NRMSE}=\frac{\mathrm{RMSE}}{{\left({q_{u}}^{A}\right)}_{\max}-{\left({q_{u}}^{A}\right)}_{\min}}\times 100$$

(10) $$(\%)\;\mathrm{MAPE}=\frac{1}{N}\sum_{b=1}^{N}\left|1-\frac{{\left({q_{u}}^{P}\right)}_{b}}{{\left({q_{u}}^{A}\right)}_{b}}\right|\times 100$$

where *q*_u_^A^ = actual peak UC strength, as presented in Figure 5; *q*_u_^P^ = predicted peak UC strength; *b* = index of summation; and *N* = number of experimental data points used for model development (*N* = 28, as outlined in Table 5).

The regression analysis outputs with respect to Equation (7) are summarized in Table 6. The high R^2^ (=0.964) and low RMSE (=17.28 kPa), NRMSE (=4.78%) or MAPE (=6.19%) values warrant a strong agreement between actual and predicted peak UC strength data. The R^2^ index merely surpassed 0.95, thus indicating that leastwise 95% of the variations in experimental observations are captured and further explained by the proposed regression model. The NRMSE index was found to be slightly less than 5%, thus signifying a maximum offset of 5% associated with the predictions. However, the *P*-value associated with some of the regression components, namely *S*_c_, *T*_c_, *S*_c_^2^, *T*_c_^2^ and *F*_c_*T*_c_, was found to be greater than 5%, implying that these components are statistically-insignificant and hence make no or little contribution towards the predictions. Statistically-insignificant terms can be eliminated to accommodate the derivation of a simplified model with unanimously-significant regression components [59]. As such, Equation (7) can be simplified as:

(11) $$q_{u}=\beta_{0}+\beta_{1}F_{c}+\beta_{4}{F_{c}}^{2}+\beta_{7}F_{c}S_{c}+\beta_{9}S_{c}T_{c}$$

**Figure 11:**
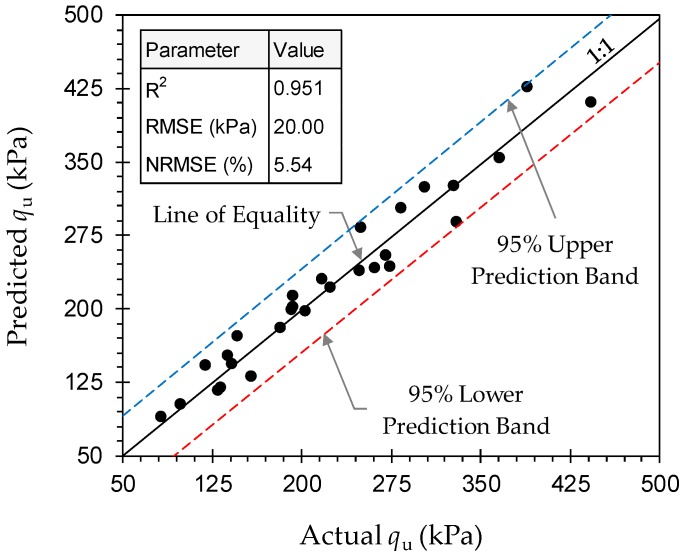
Variations of predicted, by Equation (11), against actual peak UC strength data for various JF + GBFS blends.

The regression analysis outputs with respect to Equation (11) are summarized in Table 7. The simplified model proposed in Equation (11) resulted in R^2^ = 0.951, RMSE = 20.00 kPa, NRMSE = 5.54% and MAPE = 7.28%, which are on par with that observed for Equation (7). In essence, Equation (11) suggests a more practical path towards predicting the peak UC strength while maintaining a performance similar to that offered by the more complex Equation (7). Moreover, the *p*-values associated with all of the regression components were unanimously less than 5% (see Regression Outputs in Table 7), thus corroborating their statistical significance (and contribution) towards the predictions. Figure 11 illustrates the variations of predicted, by Equation (11), against actual peak UC strength data, along with the corresponding 95% prediction bands/intervals, for various JF + GBFS blends. Despite the existence of some scatter, all data points cluster around the line of equality and firmly position themselves between the 95% upper and 95% lower prediction bands, thereby indicating no particular outliers associated with the predictions. The proposed regression model given in Equation (11) contains a total of four fitting parameters, i.e., *β*_1_, *β*_4_, *β*_7_ and *β*_9_ (*β*_0_ is equal to the peak UC strength of the natural soil), all of which can be calibrated by little experimental effort, as well as simple explicit calculations, and hence implemented for preliminary design assessments, predictive purposes and/or JF + GBFS optimization studies. Assuming that the peak UC strength of the natural soil (or *β*_0_) is at hand, the four fitting parameters can be adequately calibrated by a total of four UC tests carried out on four arbitrary JF + GBFS mix designs.

### 5.2. Sensitivity Analysis

The partial derivative sensitivity analysis technique, as commonly adopted in the literature [43,82,84], was carried out on Equation (11) to quantify the relative impacts of the independent variables, namely *F*_c_, *S*_c_ and *T*_c_, on the dependent variable *q*_u_. The overall relative impact, both positive and negative, of an independent variable, i.e., *x_a_* = *F*_c_, *S*_c_ or *T*_c_, on the dependent variable *q*_u_, commonly referred to as sensitivity, can be defined as:

(12) $$S(x_{a})=\frac{\sigma (x_{a})}{N\sigma (q_{u})}\times \sum_{b=1}^{N}\left|D_{ab}\right|∋D_{a}=\frac{dq_{u}}{dx_{a}}$$

where *D_a_* = partial derivative of *q*_u_ or Equation (11) with respect to *x_a_* = *F*_c_, *S*_c_ or *T*_c_; *σ*(*x_a_*) = standard deviation of *x_a_* data; *σ*(*q*_u_) = standard deviation of predicted *q*_u_ data; *b* = index of summation; and *N* = number of observations (*N* = 28, as outlined in Table 5).

The partial derivative term, *D_a_* = *dq*_u_/*dx_a_* in Equation (12), measures the likelihood of *q*_u_ increasing or decreasing as a result of an increase in *x_a_*. As such, the likelihood of increase or decrease in *q*_u_ as a result of an increase in *x_a_* can be, respectively, defined as:

(13) $$(\%)\;P_{P}(x_{a})=\frac{M_{P}(x_{a})}{N}\times 100$$

(14) $$(\%)\;P_{N}(x_{a})=\frac{M_{N}(x_{a})}{N}\times 100$$

where *M*_P_(*x_a_*) = number of observations where *D_a_* ≥ 0; and *M*_N_(*x_a_*) = number of observations where *D_a_* < 0.

The positive and negative impacts of an independent variable, i.e., *x_a_* = *F*_c_, *S*_c_ or *T*_c_, on the dependent variable *q*_u_ can be, respectively, defined as:

(15) $$\forall x_{a}∋D_{a}\geq 0\;,\;S_{P}(x_{a})=\frac{\sigma (x_{a})}{N\sigma (q_{u})}\times \sum_{b=1}^{N}\left|D_{ab}\right|\;∋\;D_{a}=\frac{dq_{u}}{dx_{a}}$$

(16) $$\forall x_{a}∋D_{a}<0\;,\;S_{N}(x_{a})=\frac{\sigma (x_{a})}{N\sigma (q_{u})}\times \sum_{b=1}^{N}\left|D_{ab}\right|\;∋\;D_{a}=\frac{dq_{u}}{dx_{a}}$$

It should be noted that *S*_P_(*x_a_*) and *S*_N_(*x_a_*) are, respectively, positive and negative fractions of the sensitivity parameter, *S*(*x_a_*) or Equation (12), meaning that for any given *x_a_*, *S*(*x_a_*) = *S*_P_(*x_a_*) + *S*_N_(*x_a_*).

The principal objective of any introduced soil stabilization scheme is to accommodate an increase in the peak UC strength, and as such, the variations of the positive-sensitivity parameter, *S*_P_(*x_a_*) or Equation (15), is of interest for further analysis. The positive-sensitivity parameter can be expressed in terms of percentage to facilitate a more practical comparison between the independent variables [84]:

(17) $$(\%)\;F_{P}(x_{a})=\frac{S_{P}(x_{a})}{\sum_{a=1}^{K}S_{P}(x_{a})}\times 100$$

where *F*_P_(*x_a_*) = positive contribution offered by an increase in *x_a_* resulting in an increase in *q*_u_ (in %); and *K* = number of independent variables (*K* = 3, namely *F*_c_, *S*_c_ and *T*_c_).

The sensitivity analysis results with respect to Equation (11) are summarized in Table 8. The likelihood of increase in the peak UC strength as a result of an increase in JF content was found to be 71%, thus indicating that JF-reinforcement, where 0.5% ≤ *F*_c_ ≤ 1.5%, exhibits favorable improvements only up to a particular/optimum fiber content, beyond of which marginal improvements or adverse effects, owing to fiber-clustering, can be expected (see the discussions in Section 4.3). As for GBFS content and curing time, the likelihood of increase was found to be 100% for both variables, thus indicting that GBFS-treatment, where 3% ≤ *S*_c_ ≤ 9%, consistently leads to favorable improvements which can be further enhanced by means of curing. The positive contribution offered by an increase in JF content resulting in an increase in the peak UC strength was obtained as 35%. For GBFS content and curing time, however, the positive contribution was found to be 38% and 27%, respectively. These results imply that for a given JF + GBFS blend without curing, *F*_c_ and *S*_c_ would theoretically portray an equally-significant role towards strength development. With curing, however, the overall contribution offered by GBFS-treatment profoundly outweighs that of JF-reinforcement, as *F*_P_(*S*_c_) + *F*_P_(*T*_c_) = 65% >> *F*_P_(*F*_c_) = 35%.

## 6. Conclusions

The following conclusions can be drawn from this study:

- For any given GBFS content and curing time, the greater the JF content the higher the developed strength and stiffness up to *F*_c_ = 1%; beyond 1% JF, the effect of JF-reinforcement adversely influenced the development of strength and stiffness. The composite’s ductility and toughness, however, were consistently in favor of JF-reinforcement, meaning that the greater the JF content the higher the developed ductility and toughness.
- For any given JF content, the greater the GBFS content and/or the longer the curing period, the higher the developed strength, stiffness and toughness, following monotonically-increasing trends. The composite’s ductility, however, was adversely influenced by GBFS-treatment, meaning that the greater the GBFS content and/or the longer the curing period, the lower the developed ductility.
- The addition of GBFS to JF-reinforced samples improved the soil–fiber connection interface or bonding, as the fiber units became fully embedded between and within the soil aggregates; this in turn led to a further improvement in the composite’s strength and stiffness. The ASTM D4609–08 strength criterion was used to assess the efficiency and hence applicability of the proposed JF + GBFS mix designs. In this regard, the sample *F*_1.0_*S*_9_*T*_28_ managed to satisfy ASTM’s criterion and hence can be taken as the optimum design choice.
- A non-linear, multivariable regression model was developed to quantify the peak UC strength *q*_u_ as a function of the composite’s basic index properties, i.e., JF content *F*_c_, GBFS content *S*_c_, and curing time *T*_c_. The predictive capacity of the suggested model was examined and further validated by statistical techniques. A sensitivity analysis was also carried out to quantify the relative impacts of the independent regression variables, namely *F*_c_, *S*_c_ and *T*_c_, on the dependent variable *q*_u_. The proposed regression model contained a limited number of fitting parameters, all of which can be calibrated by little experimental effort, as well as simple explicit calculations, and hence implemented for preliminary design assessments, predictive purposes and/or JF + GBFS optimization studies.

## Figures and Tables

**Table 1:** Physical and mechanical properties of K, GM and MC.

Properties	K	GM	MC	Standard Designation
Specific gravity of solids, *G*_s_	2.69	2.80	2.73	ASTM D854–14
Clay fraction [<2 μm] (%)	51	-	39	ASTM D422–07
Silt fraction [2–75 μm] (%)	48	-	55	ASTM D422–07
Fines fraction [<75 μm] (%)	99	93	94	ASTM D422–07
Sand fraction [0.075–4.75 mm] (%)	1	7	6	ASTM D422–07
Natural water content, *w*_n_ (%)	2.14	0.41	1.67	ASTM D2216–10
Liquid limit, LL (%)	44.67	-	48.67	AS 1289.3.9.1–15
Plastic limit, PL (%)	23.72	-	36.94	AS 1289.3.2.1–09
Plasticity index, PI (%)	20.95	-	11.28	AS 1289.3.3.1–09
Linear shrinkage, LS (%)	7.06	-	8.84	AS 1289.3.4.1–08
Shrinkage index, SI (%) ^1^	37.61	-	39.83	Sridharan and Nagaraj [65]
USCS classification	CI ^2^	-	MI ^3^	ASTM D2487–11
Optimum water content, *w*_opt_ (%)	19.84	-	23.52	ASTM D698–12
Maximum dry density, *ρ*_dmax_ (g/cm^3^)	1.63	-	1.56	ASTM D698–12
Unconfined compression strength, *q*_u_ (kPa) ^4^	137.62	-	85.14	ASTM D2166–16
Splitting tensile strength, *q*_t_ (kPa) ^4^	21.76	-	14.62	ASTM C496–17

^1^ SI = LL–LS; ^2^ Clay with intermediate plasticity; ^3^ Silt with intermediate plasticity; and ^4^ Tested at standard Proctor optimum condition.

**Table 2:** Chemical compositions of K and GM (as supplied by the manufacturers).

Properties	K	GM
SiO_2_ (%)	64.9	49.5
Al_2_O_3_ (%)	22.2	29.2
K_2_O (%)	2.7	8.9
TiO_2_ (%)	1.4	0.8
Fe_2_O_3_ (%)	1.0	4.6
MgO (%)	0.6	0.7
Na_2_O (%)	0.2	0.5
CaO (%)	0.1	0.4
Acidity, pH [20% slurry]	7.4	7.8
Oil absorption (mL/100 g)	34.0	36.0
Loss on ignition, LOI [at 1000 °C] (%)	6.5	<6
Specific surface area, SSA (m^2^/g)	11.2	5.3

**Table 3:** Physical and mechanical properties of JF (as supplied by the distributor).

Properties	Value
Specific gravity, *G*_s_	1.30–1.46
Length, *F*_L_ (mm)	15
Diameter, *F*_D_ (μm)	30–40
Aspect ratio, *F*_AR_ = *F*_L_/*F*_D_	375–500
Young’s modulus (GPa)	10–30
Tensile strength (MPa)	400–900
Tensile elongation at break (%)	1.5–1.8
Water absorption (%)	12

**Table 4:** Physical properties and chemical composition of GBFS (as supplied by the manufacturer).

Properties	Value
Specific gravity of solids, *G*_s_	2.87
Fines fraction [<75 μm] (%)	96
Sand fraction [0.075–4.75 mm] (%)	4
Natural water content, *w*_n_ (%)	<1
Acidity, pH [20% slurry]	9.6
Loss on ignition, LOI [at 1000 °C] (%)	<3
Specific surface area, SSA (m^2^/g)	0.7
CaO (%)	44.7
SiO_2_ (%)	27.1
Al_2_O_3_ (%)	13.6
MgO (%)	5.1
Fe_2_O_3_ (%)	3.5
TiO_2_ (%)	1.7
K_2_O (%)	0.7
Na_2_O (%)	0.2

**Table 5:** Mix designs and their properties.

Group	Designation	JF Content (%)	GBFS Content (%)
Control ^1^	*F*_0_ *S*_0_ *T*_0_	0	0
JF-reinforced	*F*_0.5_ *S*_0_ *T*_0_	0.5	0
	*F*_1.0_ *S*_0_ *T*_0_	1.0	0
	*F*_1.5_ *S*_0_ *T*_0_	1.5	0
GBFS-treated	*F*_0_ *S*_3_ *T*_7,28_	0	3
	*F*_0_ *S*_6_ *T*_7,28_	0	6
	*F*_0_ *S*_9_ *T*_7,28_	0	9
JF + GBFS	*F*_0.5_ *S*_3_ *T*_7,28_	0.5	3
	*F*_1.0_ *S*_3_ *T*_7,28_	1.0	3
	*F*_1.5_ *S*_3_ *T*_7,28_	1.5	3
	*F*_0.5_ *S*_6_ *T*_7,28_	0.5	6
	*F*_1.0_ *S*_6_ *T*_7,28_	1.0	6
	*F*_1.5_ *S*_6_ *T*_7,28_	1.5	6
	*F*_0.5_ *S*_9_ *T*_7,28_	0.5	9
	*F*_1.0_ *S*_9_ *T*_7,28_	1.0	9
	*F*_1.5_ *S*_9_ *T*_7,28_	1.5	9

^1^ Natural soil.

**Table:** **Fit-Measure** **Indices**

R ^1^	R^2^	Adjusted R^2^	RMSE (kPa)	NRMSE (%)	MAPE (%)
0.982	0.964	0.946	17.28	4.78	6.19

^1^ Coefficient of correlation.

**Table:** **Analysis** **of Variance (ANOVA)**

Source of Variation	DF ^1^	SS ^2^	MS ^3^	*F*-Value	Significance *F*
Regression	9	2.20 × 10^5^	2.44 × 10^4^	52.62	4.26 × 10^−11^ < 5% (S)
Residual	18	8.36 × 10^3^	4.64 × 10^2^		
Total	27	2.28 × 10^5^			

^1^ Degree of freedom; ^2^ Sum of squares; ^3^ Mean squares; and (S) = Significant.

**Table:** **Regression** **Outputs**

Variable	Coefficient	SE ^1^	*t*-Value	*p*-Value
Intercept	*β*_0_ = 64.75	16.19	4.00	8.42 × 10^−4^ < 5% (S)
*F*_c_	*β*_1_ = 171.31	28.76	5.96	1.23 × 10^−5^ < 5% (S)
*S*_c_	*β*_2_ = 2.43	13.06	0.19	8.55 × 10^−1^ > 5% (NS)
*T*_c_	*β*_3_ = 1.48	6.68	0.22	8.27 × 10^−1^ > 5% (NS)
*F*_c _^2^	*β*_4_ = −85.99	16.29	−5.28	5.10 × 10^−5^ < 5% (S)
*S*_c _^2^	*β*_5_ = 0.26	1.04	0.25	8.02 × 10^−1^ > 5% (NS)
*T*_c _^2^	*β*_6_ = −0.04	0.20	−0.22	8.31 × 10^−1^ > 5% (NS)
*F*_c_ × *S*_c_	*β*_7_ = 6.65	2.53	2.63	1.70 × 10^−2^ < 5% (S)
*F*_c_ × *T*_c_	*β*_8_ = −0.17	0.68	−0.25	8.09 × 10^−1^ > 5% (NS)
*S*_c_ × *T*_c_	*β*_9_ = 0.61	0.17	3.55	2.28 × 10^−3^ < 5% (S)

^1^ Standard error; (S) = Significant; and (NS) = Not Significant.

**Table:** **Fit-Measure** **Indices**

R ^1^	R^2^	Adjusted R^2^	RMSE (kPa)	NRMSE (%)	MAPE (%)
0.976	0.951	0.943	20.00	5.54	7.28

^1^ Coefficient of correlation.

**Table:** **Analysis** **of Variance (ANOVA)**

Source of Variation	DF ^1^	SS ^2^	MS ^3^	*F*-Value	Significance *F*
Regression	4	2.17 × 10^5^	5.43 × 10^4^	111.49	1.04 × 10^−14^ < 5% (S)
Residual	23	1.12 × 10^4^	4.87 × 10^2^		
Total	27	2.28 × 10^5^			

^1^ Degree of freedom; ^2^ Sum of squares; ^3^ Mean squares; and (S) = Significant.

**Table:** **Regression** **Outputs**

Variable	Coefficient	SE ^1^	*t*-Value	*P*-Value
Intercept	*β*_0_ = 89.14	9.70	9.19	3.69 × 10^–9^ < 5% (S)
*F*_c_ (%)	*β*_1_ = 148.90	27.51	5.41	1.69 × 10^–5^ < 5% (S)
*F*_c _^2^	*β*_4_ = −85.99	16.68	−5.16	3.17 × 10^–5^ < 5% (S)
*F*_c_ × *S*_c_	*β*_7_ = 10.52	1.69	6.22	2.40 × 10^–6^ < 5% (S)
*S*_c_ × *T*_c_	*β*_9_ = 0.65	0.06	11.08	1.07 × 10^–10^ < 5% (S)

^1^ Standard error; and (S) = Significant.

**Table 8:** Summary of the sensitivity analysis results with respect to Equation (11).

Variable, *x_a_*	*D*_a_ = *dq*_u_/*dx*_a_	*S*(*x_a_*)	*P*_P_(*x_a_*) (%)	*P*_N_(*x_a_*) (%)	*S*_P_(*x_a_*)	*S*_N_(*x_a_*)	*F*_P_(*x_a_*) (%)
JF content, *F*_c_	$\beta_{1}+2\beta_{4}F_{c}+\beta_{7}S_{c}$	0.639	71	29	0.548	0.090	35
GBFS content, *S*_c_	$\beta_{7}F_{c}+\beta_{9}T_{c}$	0.605	100	0	0.605	0	38
Curing time, *T*_c_	$\beta_{9}S_{c}$	0.427	100	0	0.427	0	27
